# Temporal dynamics from phosphoproteomics using endoscopic biopsy specimens provides new therapeutic targets in stage IV gastric cancer

**DOI:** 10.1038/s41598-022-08430-7

**Published:** 2022-03-25

**Authors:** Hidekazu Hirano, Yuichi Abe, Yosui Nojima, Masahiko Aoki, Hirokazu Shoji, Junko Isoyama, Kazufumi Honda, Narikazu Boku, Kenji Mizuguchi, Takeshi Tomonaga, Jun Adachi

**Affiliations:** 1 Laboratory of Proteome Research, National Institute of Biomedical Innovation, Health and Nutrition, Osaka, 567-0085 Japan; 2 Laboratory of Proteomics for Drug Discovery, Center for Drug Design Research, National Institute of Biomedical Innovation, Health and Nutrition, Osaka, 567-0085 Japan; 3 Gastrointestinal Medical Oncology Division, National Cancer Center Hospital, Tokyo, 104-0045 Japan; 4 Department of Medicine, Keio University Graduate School of Medicine, Tokyo, 160-8582 Japan; 5 Department of Biomarkers for Early Detection of Cancer, National Cancer Center Research Institute, Tokyo, 104-0045 Japan; 6 Department of Bioregulation, Nippon Medical School, Bunkyo-ku, Tokyo, 113-8602 Japan; 7 Laboratory of Bioinformatics, Artificial Intelligence Center for Health and Biomedical Research (ArCHER), National Institutes of Biomedical Innovation, Health and Nutrition, Osaka, 567-0085 Japan; 8 Center for Mathematical Modeling and Data Science, Osaka University, Osaka, 560-8531 Japan; 9 Institute for Protein Research, Osaka University, Osaka, 565-0871 Japan; 10 Laboratory of Clinical and Analytical Chemistry, Center for Drug Design Research, National Institute of Biomedical Innovation, Health and Nutrition, Osaka, 567-0085 Japan; 11 Kyoto Innovation Center for Next Generation Clinical Trials and iPS Cell Therapy (Ki-CONNECT), Kyoto University Hospital, Kyoto, 606-8507 Japan; 12 Department of Medical Oncology and General Medicine, IMSUT Hospital, Institute of Medical Science, University of Tokyo, Tokyo, 108-8639 Japan; 13 Division of Molecular Diagnostics, Aichi Cancer Center Research Institute, Nagoya, 464-8681 Japan

**Keywords:** Gastric cancer, Mass spectrometry, Proteome

## Abstract

Phosphoproteomic analysis expands our understanding of cancer biology. However, the feasibility of phosphoproteomic analysis using endoscopically collected tumor samples, especially with regards to dynamic changes upon drug treatment, remains unknown in stage IV gastric cancer. Here, we conducted a phosphoproteomic analysis using paired endoscopic biopsy specimens of pre- and post-treatment tumors (Ts) and non-tumor adjacent tissues (NATs) obtained from 4 HER2-positive gastric cancer patients who received trastuzumab-based treatment and from pre-treatment Ts and NATs of 4 HER2-negative gastric cancer patients. Our analysis identified 14,622 class 1 phosphosites with 12,749 quantified phosphosites and revealed molecular changes by HER2 positivity and treatment. An inhibitory signature of the ErbB signaling was observed in the post-treatment HER2-positive T group compared with the pre-treatment HER2-positive T group. Phosphoproteomic profiles obtained by a case-by-case review using paired pre- and post-treatment HER2-positive T could be utilized to discover predictive or resistant biomarkers. Furthermore, these data nominated therapeutic kinase targets which were exclusively activated in the patient unresponded to the treatment. The present study suggests that a phosphoproteomic analysis of endoscopic biopsy specimens provides information on dynamic molecular changes which can individually characterize biologic features upon drug treatment and identify therapeutic targets in stage IV gastric cancer.

## Introduction

Gastric cancer is the third leading cause of cancer-related death worldwide^1^. Over the past two decades, the therapeutic landscape for advanced gastric cancer has changed significantly with the approval of new agents. Trastuzumab and trastuzumab deruxtecan used as the standard-of-care in human epidermal growth factor receptor 2 (HER2)-positive gastric cancer, which accounts for 20% of all gastric cancers^2,3^. Based on the results of phase 3 trials, fluoropyrimidine plus platinum with or without nivolumab (immune checkpoint inhibitor) is the standard-of-care for HER2-negative advanced gastric cancer in the first-line setting and fluoropyrimidine plus platinum with trastuzumab for HER2-positive advanced gastric cancer^2,4,5^. However, the majority of patients experience disease progression during the treatment and there remains an unmet need for more effective therapeutic options for advanced gastric cancer.

The facilitation of large-scale genomic research following the advent of next generation sequencing has provided insights into the biology of gastric cancer^6,7^. Concurrently, comprehensive genomic profiling has been implemented for personalization of therapeutic options in clinical practice. Despite the introduction of precision medicine, only a small fraction of patients has received genomically-matched treatments^8,9^. In gastric cancer, the scarcity of targetable genetic alterations is an important feature^10^. Therefore, new approaches for identifying biological features beyond genomic characterization are required to make additional breakthroughs in personalized medicine. In this regard, correlating proteomic profiles with specific cancer phenotypes has the potential to expand the therapeutic horizon in advanced gastric cancer.

Phosphorylation is one of the most common posttranslational modifications and regulates many biological processes including oncogenesis. The Clinical Proteomic Tumor Analysis Consortium performed proteomic/phosphoproteomic characterization of multiple types of solid tumors, and proposed that phosphoproteomic analysis could be used to identify therapeutic targets^11–19^. In addition, high-sensitive phosphoproteomic analyses have been performed using cancer cell lines and surgical specimens^20,21^. For patients with metastatic cancer treated with drug therapy, it is required to obtain tumor tissues for application of phosphoproteomic analysis on demand (e.g., at disease progression), which limits the type of clinical specimens. It is highly invasive to perform surgical resection of tumors with the intent for tissue collection in patients with stage IV gastric cancer. Furthermore, phosphoproteomic status changes rapidly and can be affected by ischemia during surgical procedures^22^. These issues hamper the clinical applicability of phosphoproteomic analysis in patients with metastatic disease. To overcome this problem, we have developed a highly sensitive phosphoproteomic analysis platform using fresh-frozen endoscopic biopsy specimens containing a small amount of protein (approximately 300 µg/specimen)^23^. Using this platform, we performed kinase-substrate enrichment analysis of phosphoproteomic data using paired gastric cancer tissues and normal gastric tissues at static condition and found that cell cycle-related kinases and DNA damage response signals were activated in the cancer. Furthermore, the kinase activity profiles of tumors differed greatly among individuals.

Although molecular targeted agents can elicit an impressive initial response, intrinsic and acquired drug resistance is a relevant problem, dampening long-term disease control. Serial biopsies after administration of molecular targeted agents can reveal changes in activation or inhibition of intracellular signaling pathways, thereby helping to identify resistance mechanisms in individual patients. In this context, phosphoproteomic analysis of serial endoscopic biopsy specimens during treatment or upon progression, may facilitate the elucidation of resistance mechanisms and the discovery of therapeutic targets. However, the feasibility and molecular significance of phosphoproteomic analysis using tumor specimens collected sequentially remain unknown.

Here, we conducted a phosphoproteomic analysis using endoscopic biopsy specimens obtained from stage IV gastric cancer patients. Especially, we investigated the dynamic molecular changes after trastuzumab-containing therapy in HER2-positive gastric cancer using paired pre- and post-treatment tumor specimens for evaluating the utility of our method to understand the effect of drug therapy involving a molecular targeted agent from the phosphoproteomic landscape. In addition, we estimated the spectrum of active kinase of gastric cancer by comparing tumor specimens with non-tumor adjacent tissues on a case-by-case basis.

## Results

### Overview of phosphoproteomic analysis

**Figure 1:**
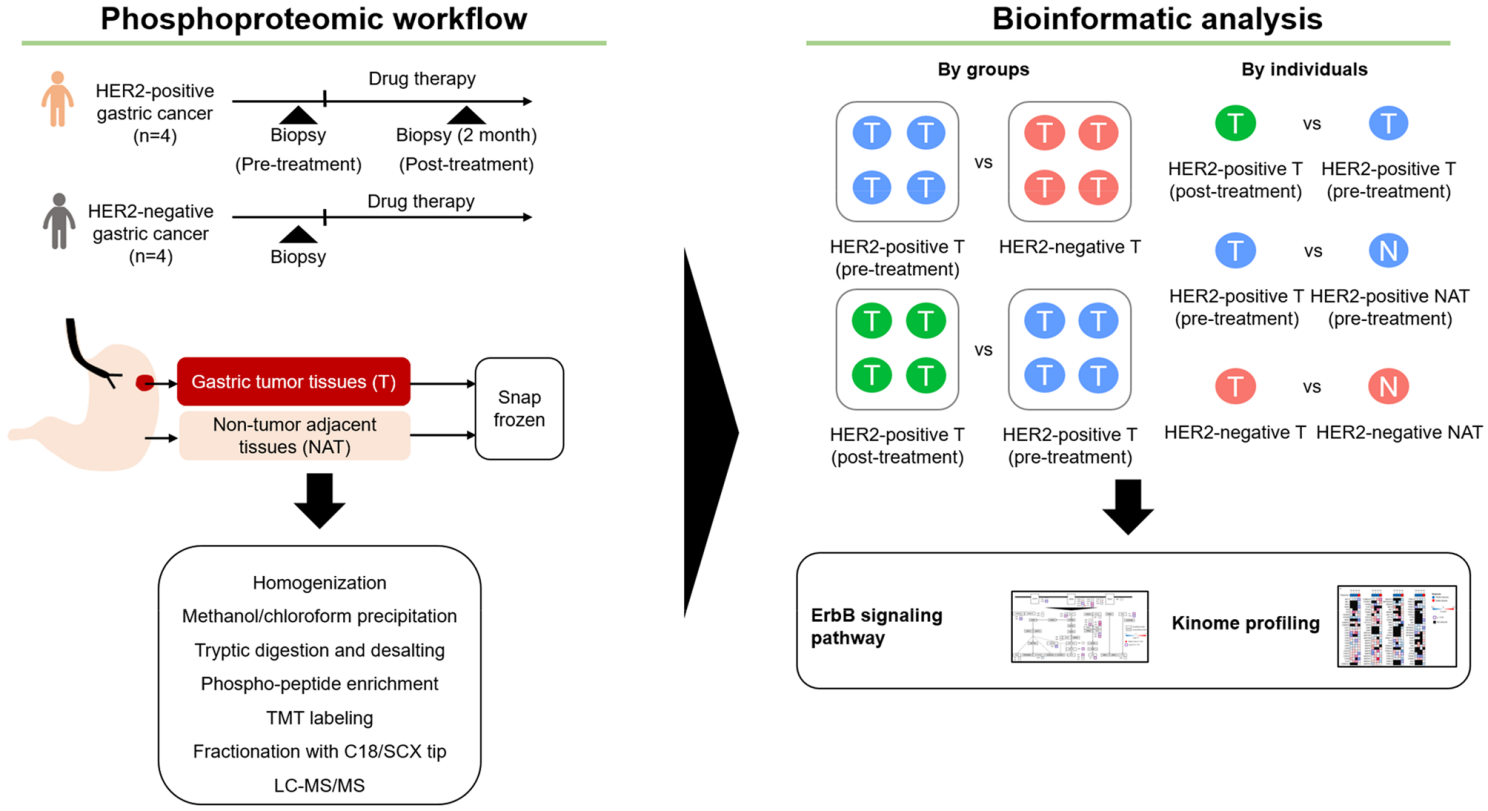
Schematic diagram of the quantitative phosphoproteomic analysis in this study. Using endoscopic procedures, tumor tissues and non-tumor adjacent tissues were collected from four patients with HER2-positive gastric cancer (before and after drug therapy [trastsuzumab-containing therapy]) and four patients with HER2-negative gastric cancer. Those specimens were subject to sample preparation for quantitative phosphoproteomic analysis. Using phosphoproteomic data, bioinformatics analyses were conducted to evaluate molecular differences by groups and by individuals.

Figure 1 shows a schematic diagram of quantitative phosphoproteomic analysis of gastric tumor tissues (Ts) and non-tumor adjacent tissues (NATs) before and after trastuzumab-based treatment of four patients with HER2-positive cancer and Ts and NATs from four patients with HER2-negative cancer (Fig. S1a).

**Figure 2:**
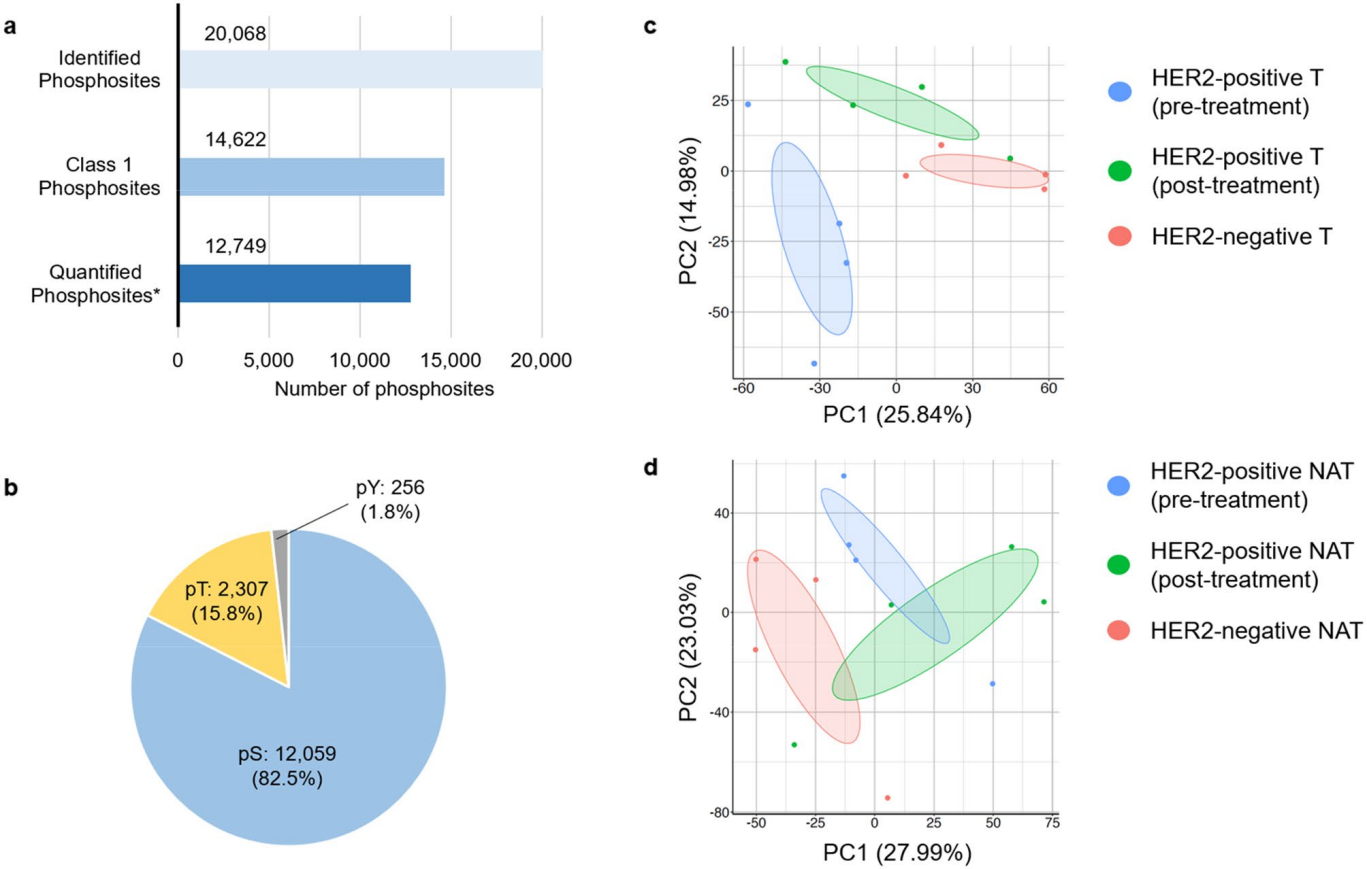
Summary of the results of phosphoproteomic analysis. (**a**) Number of identified phosphosites, class 1 phosphosites, quantified class 1 phosphosites. Asterisk (quantified phosphosites) means the phosphosites with quantitative values in at least one sample. (**b**) The proportions of phospho-serine (pS, blue), phospho-threonine (pT, yellow), phospho-tyrosine (pY, grey). (**c**) Principal component analysis of tumor samples using phosphoproteomic data. (**d**) Principal component analysis of non-tumor adjacent samples using phosphoproteomic data.

Clinical information on the patients is summarized in Table 1. With the exception of Patient 4, a partial response (PR) was elicited by drug therapy in all patients with HER2-positive gastric cancer. In total, 14,622 class 1 phosphosites including 12,059 phospho-serine sites (82.5%), 2,307 phospho-threonine sites (15.8%), and 256 phospho-tyrosine sites (1.8%) were identified from the phosphoproteomic data (Fig. 2a,b). Of the class 1 phosphosites, 12,749 phosphosites (87.2%) had quantitative values in at least one sample (Fig. 2a). Of the quantified phosphosites, the percentage of phosphosites that showed quantitative values in all three groups (cell line mixture, primary tumor tissue, and non-tumor adjacent tissue) was 97.6% and the percentage that showed quantitative values only in the cell line mixture samples was 0.6% (Fig. S1b). In total, 4,986 proteins were identified from global proteomic analysis. Of quantified protein in at least one sample (4,809 proteins), the percentage of proteins that showed quantitative values in all three groups (cell line mixture, primary tumor tissue, and non-tumor adjacent tissue) was 99.7% and the percentage that showed quantitative values only in the cell line mixture samples was 0% (Fig. S1c).

**Table 1:** Summary of clinical information on the patients for the phosphoproteomic analysis. Best overall response was defined according to the Response Evaluation Criteria in Solid Tumors (RECIST) version 1.1.

Patient	Age	Sex	Metastatic site	Stage	Histology	Prior treatment	Treatment	BOR
HER2-positive 1	59	Male	LYM, HEP, PER	IV	Intestinal	None	SOX + Tmab	PR
HER2-positive 2	44	Female	LYM, PER	IV	Diffuse	None	XP + Tmab	PR
HER2-positive 3	66	Female	LYM, HEP	IV	Intestinal	None	XP + Tmab	PR
HER2-positive 4	64	Male	LYM, HEP, PER, PUL	IV	Diffuse	None	XP + Tmab	SD
HER2-negative 1	72	Male	LYM, HEP	IV	Intestinal	Yes		
HER2-negative 2	68	Male	LYM, HEP, PER	IV	Intestinal	None		
HER2-negative 3	71	Female	LYM, PER	IV	Diffuse	None		
HER2-negative 4	64	Female	LYM, PER, PLU	IV	Diffuse	None		

*BOR* best overall response, *HEP* liver, *HER2* human epidermal growth factor receptor 2, *LYM* lymph node, *PER* peritoneum, *PLU* pleura, *PR* partial response, *PUL* lung, *SD* stable disease, *SOX* S-1 plus oxaliplatin, *Tmab* trastuzumab, *XP* capecitabine plus cisplatin.

Next, we performed principal component analysis (PCA) using batch-corrected phosphoproteomic and proteomic data (data without batch correction: Table S1 [phosphoproteomic analysis], S2 [global proteomic analysis]; data with batch correction: Table S3 [phosphoproteomic analysis], Table S4 [global proteomic analysis]). PCA using phosphoproteomic data revealed a clear separation between HER2-negative T and pre-treatment HER2-positive T (Fig. 2c). In addition, plots of post-treatment HER2-positive T were completely shifted from those of pre-treatment HER2-positive T (Fig. 2c). Taken together, these results indicate that HER2 positivity and drug treatment have clear effects on the phosphoproteomic signature. Plots of NAT samples before and after drug treatment were overlapped, indicating that drug treatment had less of an effect on the NAT phosphoproteomic profiles (Fig. 2d). PCA using global proteomic data from T or NAT samples suggested that the phosphoproteomic data separated tumors more clearly according to HER2 positivity and treatment exposure (Fig. S2a,b). Although the differences were not statistically significant, HER2 expression tended to be higher in the pre-treatment HER2-positive T group compared with the post-treatment HER2-positive T group, and with the HER2-negative T group (Fig. S2c).

### Comparison of phosphorylation status between the pre-treatment HER2-positive and the HER2-negative T group

**Figure 3:**
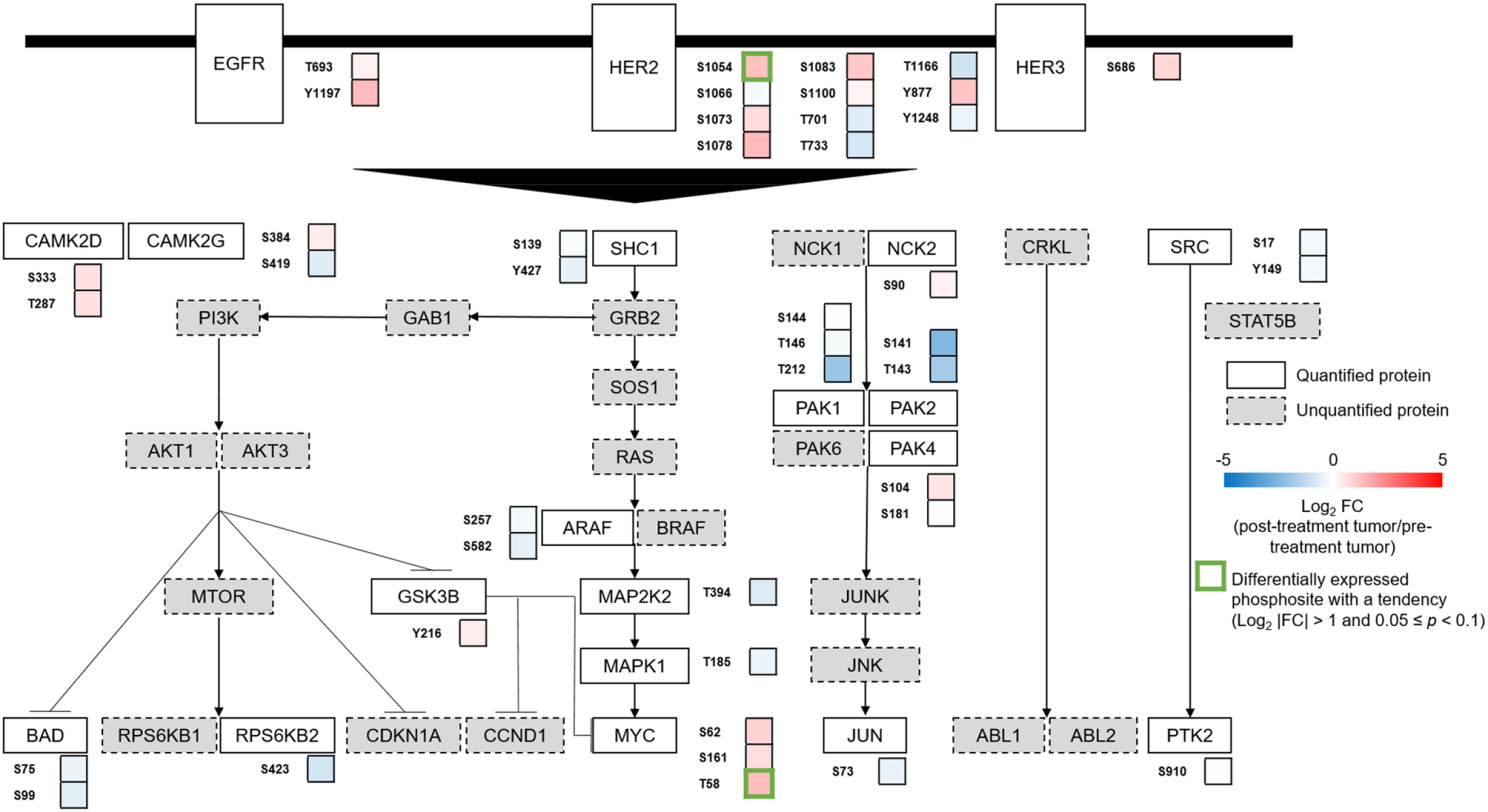
Dynamics of phosphosites in the ErbB pathway (the HER2-positive gastric cancer [pre-treatment] group versus the HER2-negative gastric cancer group). Proteins in the ErbB signaling pathway are selected from the KEGG database^65^. Proteins with quantified phosphosites are shown as white boxes with solid lines. Proteins without quantified phosphosites are shown as grey boxes with dotted lines. Differentially expressed phosphosites with significance (log_2_ |FC| > 1 and *p* < 0.05) are depicted by a purple line. Differentially expressed phosphosites with a tendency (log_2_ |FC| > 1 and and 0.05 ≤ *p* < 0.1) are depicted by a green line.

To capture unique phosphosites in HER2-positive gastric cancer, we overlaid the phosphoproteomic data from the pre-treatment HER2-positive and HER2-negative T groups with phosphosites in the ErbB signaling pathway in the Kyoto Encyclopedia of Genes and Genomes (KEGG) database (Fig. 3, Table S5). Several phosphosites in the ErbB signaling pathway were upregulated with significance (HER2 [T733]) or with a tendency (PAK2 [T143], MYC [S62]) in the HER2-positive T group. Of note, increased phosphorylation of serine 62 of MYC is indicative of the activation of this multifunctional oncoprotein, which cooperates with HER2 to stimulate cell proliferation^24,25^. Increased phosphorylation of MAP2K2 (MEK2) at threonine 394 and MAPK1 (ERK2) at threonine 185 was observed without statistical significance or tendency, which might be associated with inhibition of negative feedback of RAS/ERK signaling, resulting in HER2 (Y1248) downregulation^26,27^. These results suggest that the ErbB signaling is activated in HER2-positive gastric cancer compared with HER2-negative gastric cancer.

Then, we performed pathway enrichment analysis for differentially expressed phosphosites with significance between two groups (402 upregulated phosphosites in the pre-treatment HER2-positive T group and 478 upregulated phosphosites in the HER2-negative T group, as shown in Fig. S3a,b and Table S5). The results of pathway analysis with *p*-values < 0.05 are summarized in Table S5. Of note, the phosphosites upregulated in the HER2-positive T group were involved in lysine degradation, consistent with a previous finding by whole-genome sequencing^28^. The phosphosites upregulated in the HER2-negative T group were involved in glycerophospholipid metabolism and the p53 signaling pathway.

### Phosphoproteomic changes after drug therapy in HER2-positive gastric cancer

**Figure 4:**
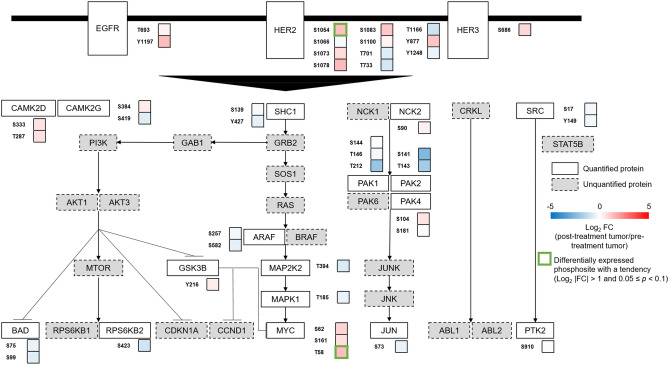
Dynamics of phosphosites in the ErbB pathway (between pre- and post-treatment the HER2-positive gastric cancer groups). Proteins in the ErbB signaling pathway are selected from the KEGG database^65^. Proteins with quantified phosphosites are shown as white boxes with solid lines. Proteins without quantified phosphosites are shown as grey boxes with dotted lines. Differentially expressed phosphosites with a tendency (log_2_ |FC| > 1 and and 0.05 ≤ *p* < 0.1) are depicted by a green line.

Next, we investigated the impact of drug therapy on the ErbB signaling pathway in the post-treatment HER2-positive T group versus the pre-treatment HER2-positive T group (Fig. 4, Table S6). An inhibitory signature of the ErbB signaling was observed without statistical significance or tendency in post-treatment HER2-positive T group, such as the downregulation of mTOR (RPS6KB2 [S6K2, (S423)]), and JUN signaling (PAK1 [T212], PAK2 [S141], JUN [S73]). Decreased levels of these phosphosites are associated with inactivation of the corresponding oncoproteins^29–36^. In addition, the decreased phosphorylation of MAP2K2 (MEK2) at threonine 394 and MAPK1 (ERK2) at threonine 185 might be explained by the inhibition of the ErbB signaling by drug therapy^26,27^. HER2 (S1054) and MYC (T58) tended to be upregulated in the post-treatment HER2-positive T group. The increased phosphorylation of the serine 1054 residue of HER2 might be due to a feedback response to anti-HER2 blockade^26^. In addition, phosphorylation of MYC at threonine 58 results in proteasomal degradation of this oncoprotein^37^. Collectively, these data capture inhibitory modifications of the ErbB signaling pathway in post-treatment HER2-positive gastric cancer.

Then, we performed pathway enrichment analysis for differentially expressed phosphosites with significance between two groups. (209 upregulated phosphosites and 135 downregulated phosphosites in the post-treatment HER2-positive T group, as shown in Fig. S4a,b and Table S6). The results of pathway analysis with *p*-values < 0.05 are summarized in Table S6. The spliceosome pathway was included among the increased activated pathways in the post-treatment HER2-positive group. Spliceosomes play a role in the alternative mRNA splicing machinery, which is induced by platinum and trastuzumab-based treatment^38,39^. In addition, the nucleotide excision repair pathway was also activated. Nucleotide excision repair is an important cellular defense mechanism against platinum agents that elicit DNA damage^40^. Taken together, these data suggest that our method provides information on phosphoproteomic changes associated with the response to drug therapy in HER2-positive gastric cancer.

### Individual variation of phosphorylation status in ErbB signaling after drug therapy in HER2-positive gastric cancer

**Figure 5:**
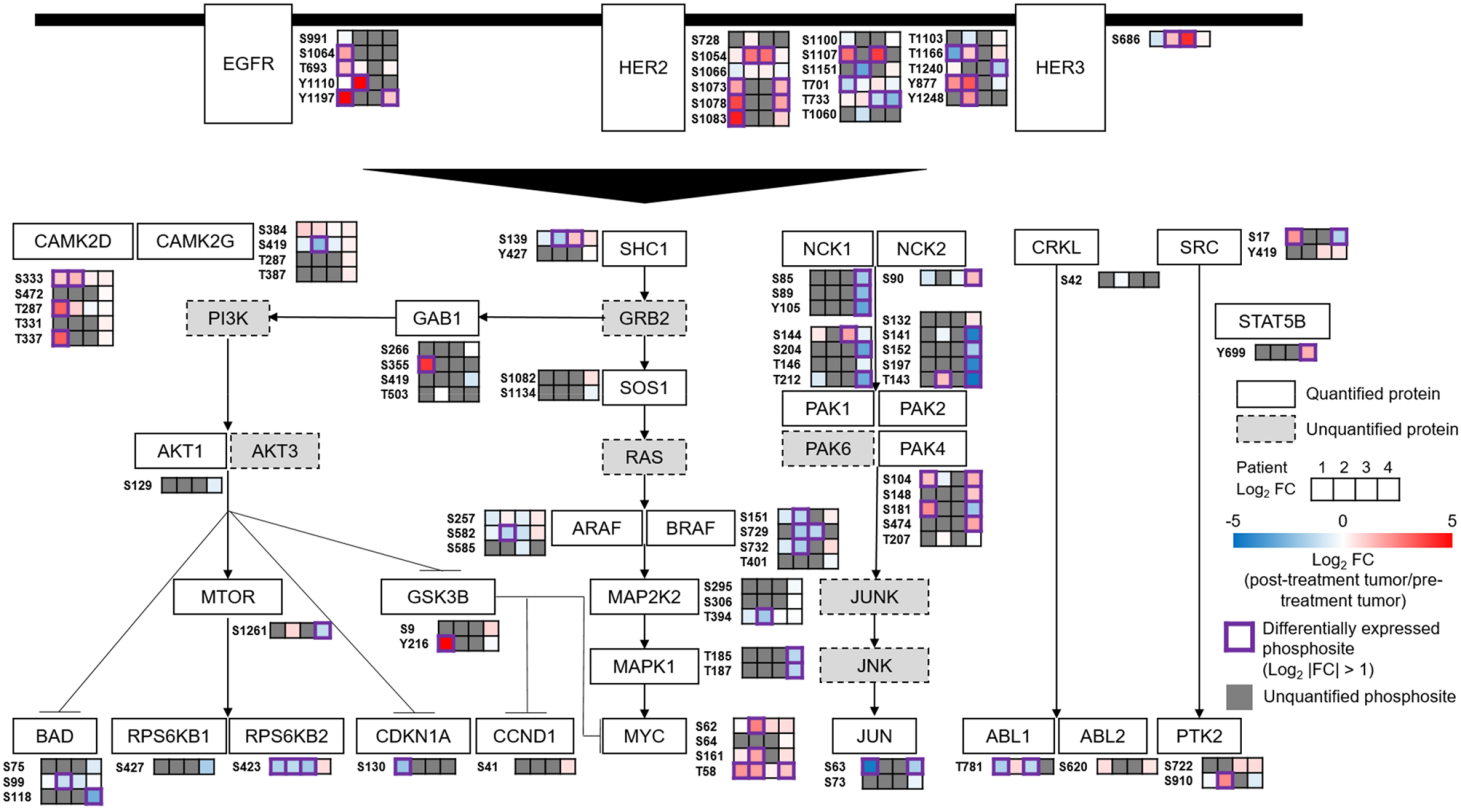
Individual comparison of phosphorylation status between pre- and post-treatment HER2-positive gastric cancer. Proteins in the ErbB signaling pathway are selected from the KEGG database^65^. Proteins with quantified phosphosites are shown as white boxes with solid lines. Proteins without quantified phosphosites are shown as grey boxes with dotted lines. Differentially expressed phosphosites (log_2_ |FC| > 1) are depicted by a purple line. Phosphosites with no quantitative value are filled with grey.

We assumed that individual phosphoproteomic data might show inter-patient variations. To capture individual variation of phosphorylation status in the ErbB signaling upon drug therapy, a case-by-case review using paired pre- and post-treatment HER2-positive T samples derived from 4 patients was performed (Fig. 5, Table S7). In this analysis, a total of 17 phosphosites on HER2 protein were quantified in at least one patient. Considering that the function of those phosphosites in tumor is largely unexplored, we focused on the downstream components of the ErbB pathway to investigate the effects anti-HER2 blockade^41^. Our data revealed that RPS6KB2 (S6K2, [S423]) and NCK2 (S90) phosphosites were upregulated only in Patient 4 (who did not experience a PR) but were downregulated in Patient 1–3 (each of whom experienced a PR). Notably, phosphorylation of serine 423 of RPS6KB2 (S6K2) leads to its activation, suggesting that this phosphosite is a predictive biomarker for the efficacy of trastuzumab-based treatment^35^. In addition, we observed the upregulation of several other phosphosites in the post-treatment tumor of Patient 4, such as PAK4 (S474) and STAT5B (Y699), potentially associated with bypass mechanisms for acquired resistance^42,43^. Among three patients who achieve a PR, complex variations in the activation were shown in the ErbB pathway. As a common feature, downregulation of several phosphosites on RAF proteins were observed exclusively in responded patients, suggesting an effect of HER2 blockade on the ErbB pathway^44^. Collectively, these phosphoproteomic data reveal distinct profiles in signaling pathways on an individual patient level and could thus be used for identifying molecules that predict the response to drug therapy or link to acquired resistance.

### Individual activated kinases after drug therapy for HER2-positive gastric cancer

**Figure 6:**
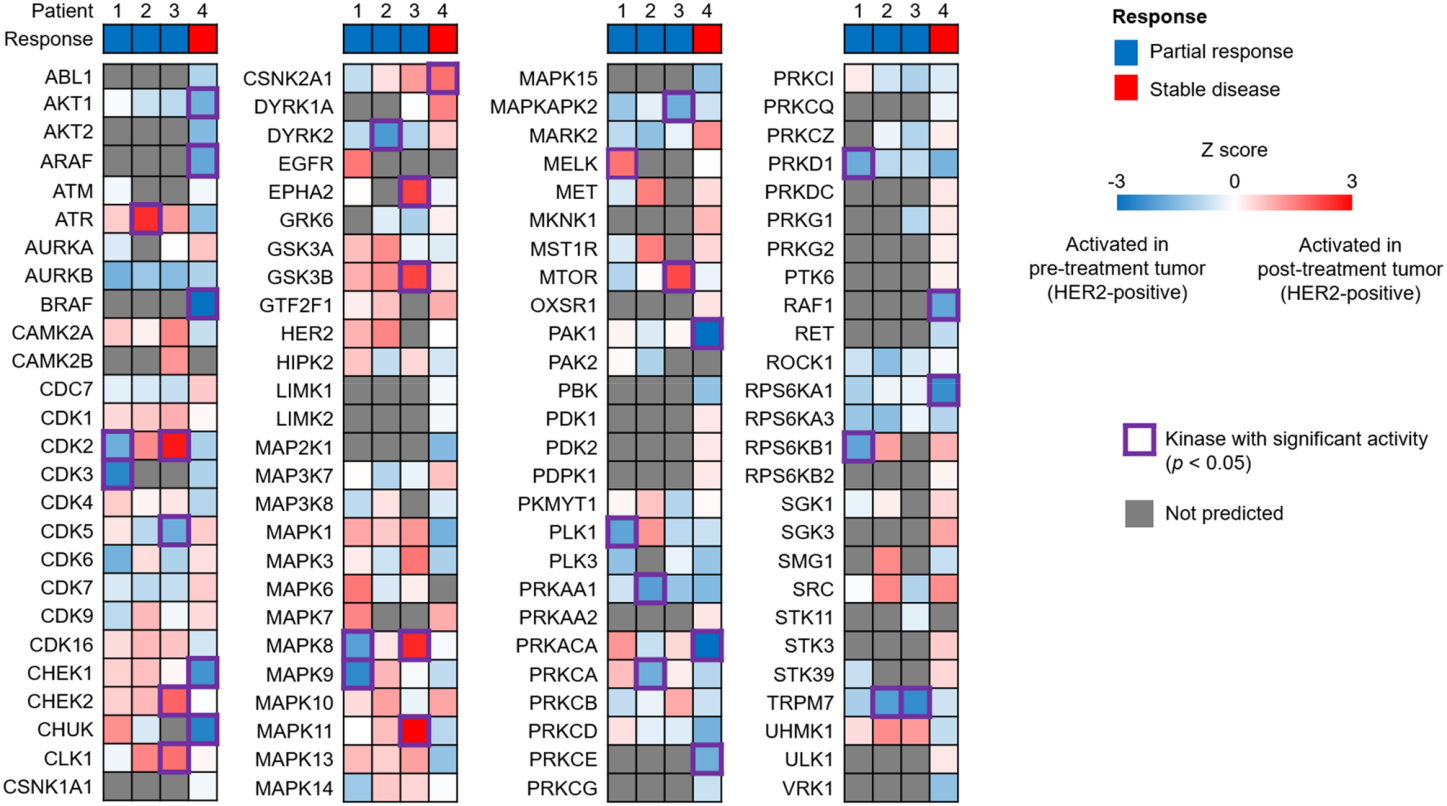
Individual kinome profiling of post-treatment HER2-positive gastric cancer in comparison with the corresponding pre-treatment HER2-positive gastric cancer. Information on the responses to drug therapy is shown at the top of the kinome profiling. Kinases with significant activity (*p* < 0.05) are depicted by purple lines. Kinases with no information are filled with grey.

Considering the clinical success in the development of kinase inhibitors, an understanding of kinome-wide dysregulation may help to individualize cancer treatment further. We subjected our individual phosphoproteomic data of HER2-positive gastric cancer to kinase-substrate enrichment analysis (KSEA) to identify active kinases after drug therapy in a personalized manner (Fig. 6, Table S8). Patient-specific profiles showed no clear difference in kinase activities between responding and non-responding patients, suggesting that the change in kinase activities in response to drug therapy was highly inter-individual. Notably, several kinases were upregulated exclusively in Patient 4 in whom PR was not achieved, including AURKA, CDC7, CDK7, DYRK1A, MAP3K7, MARK2, RPS6KB2 (S6K2), SGK3, STK3, STK39, and ULK. Activation of these kinases might be associated with acquired resistance to drug therapy and they may be candidates for therapeutic targets. Phosphoproteomic data also revealed trends of upregulation of phosphorylation at sites that activate two kinases (CDK7[T170], RPS6KB2(S6K2) [S423]) after treatment in the post-treatment tumor of Patient 4 compared with those of the other patients (Fig. S5, Table S7). Taken together, these data suggest that phosphoproteomic analysis might be used to evaluate kinase activity after drug therapy and potentially guide therapeutic strategies on an individual patient level.

### Individual activated kinases in T compared to NAT

**Figure 7:**
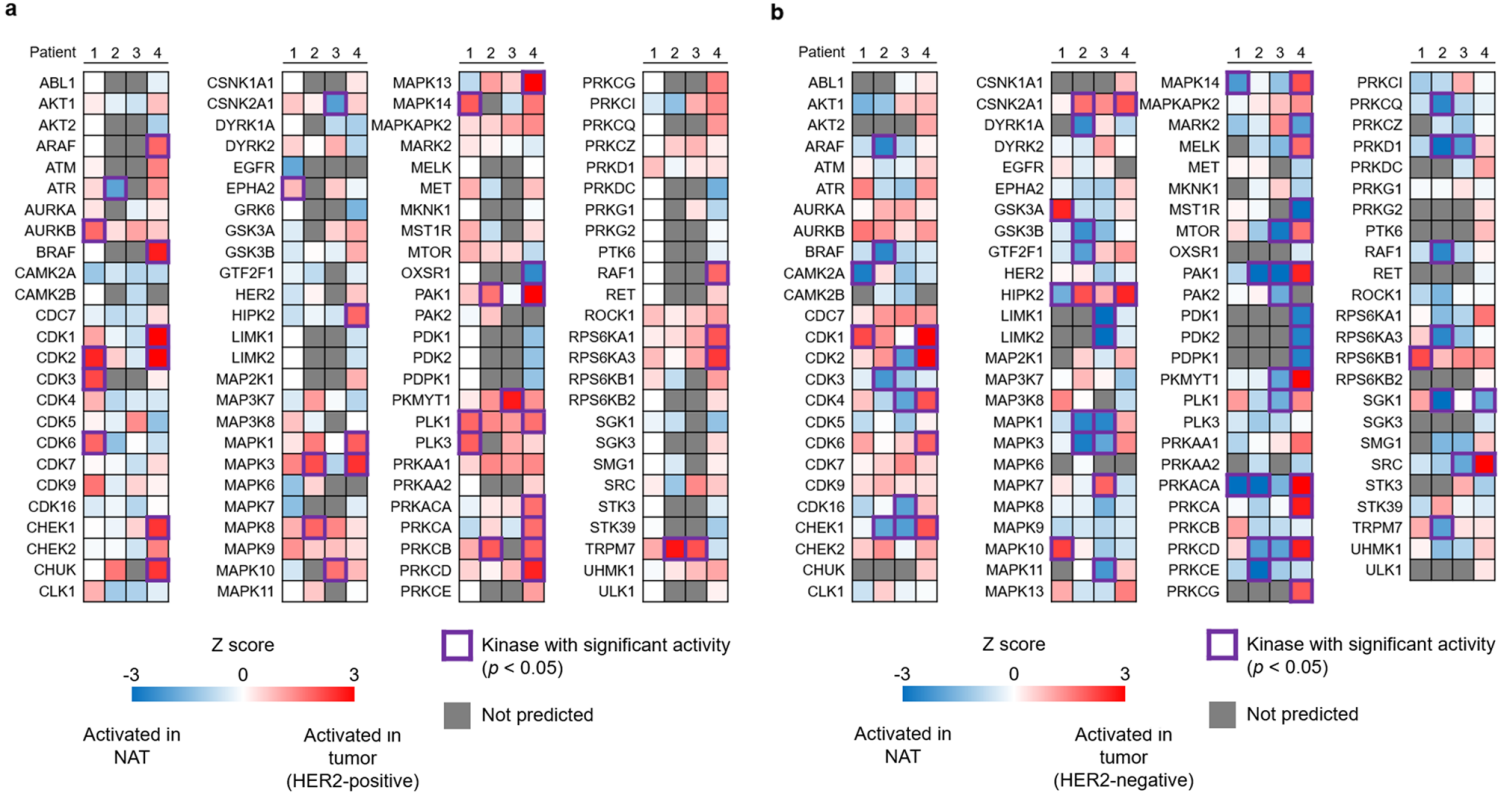
Individual kinome profiling of gastric cancer versus the corresponding non-tumor adjacent tissue. Information on the patients is shown at the top of the kinome profiling. Kinases with significant activity (*p* < 0.05) are depicted by purple lines. Kinases with no information are filled with grey. (**a**) Kinase profiles of pre-treatment HER2-positive gastric cancer. (**b**) Kinase profiles of HER2-negative gastric cancer.

Next, we exploited our analysis of individual phosphoproteomic data of T and NAT using KSEA to identify kinases that were activated in pre-treatment HER2-positive and HER2-negative gastric cancer (Fig. 7a,b, Table S9, S10). Regardless of HER2 positivity, kinases in cell cycle pathways (e.g., CDK2, CDK6) or those involved in the DNA damage response (e.g., ATM, ATR, CHEK2) were upregulated. Intriguingly, there was no signal of strong HER2 activation in pre-treatment HER2-positive gastric cancer samples, despite the increased HER2 protein level in this group (Fig. S2c), indicating the complexity of the relationship between protein abundance and functionality.

## Discussion

In this study, we performed phosphoproteomic analysis of advanced gastric cancer using endoscopic biopsy specimens. By using endoscopic specimens immediately frozen in liquid nitrogen, we minimized the ischemic effect on protein phosphorylation for obtaining physiological phosphoproteomic data. Even with limited amount of protein, we identified more than 14,000 class 1 phosphosites, allowing characterization of tumor biology not only before but also after drug therapy in metastatic disease. Distinct differences in phosphorylation status were observed between HER2-positive cancer and HER2-negative cancer, and between pre- and post-treatment HER2-positive cancer. By coupling individual phosphoproteomic data with kinase-substrate relationships, activated kinases in post-treatment tumors in comparison with pre-treatment tumors with regards to HER2-positive gastric cancer, and those in tumors in comparison with non-tumor adjacent tissues could be inferred in a personalized manner. To our knowledge, the present study firstly demonstrated the concept of investigating the dynamic molecular changes induced by drug therapy from the phosphoproteomic landscape in stage IV gastric cancer.

By comparing the phosphoproteomic data of pre- and post-treatment HER2 positive gastric cancer groups, we detected an inhibitory signature of the ErbB signaling after trastuzumab-based treatment. In addition, pathway enrichment analysis showed that particular pathways that were activated in specific groups (e.g., lysine degradation in pre-treatment HER2-positive gastric cancer compared with HER2-negative cancer; spliceosome and nucleotide excision repair pathways in the post-treatment HER2-positive gastric cancer compared with pre-treatment HER2-positive gastric cancer). A case-by-case review of comparison of pre- and post-phosphoproteomic data, a significant downregulation of RPS6KB2 (S6K2, [S423]) was observed in three patients with a PR but not in one patient with a PR. Previous researches revealed demonstrate that trastuzumab inhibits the phosphorylation of S6K^45,46^. In addition, trastuzumab-based treatment requires the inhibition of S6K phosphorylation to enhance fluorouracil-induced apoptosis in gastric cancer cell lines with HER2 amplification^45^. Considering that activation of RPS6KB2 (S6K2) is accompanied by phospholylation of specific sites such as S423, our data indicate that the phosphorylation level of RPS6KB2 (S6K2, [S423]) immediately after administration of treatment can be utilized as a predictive marker for the response to trastuzumab-based treatment^47^. Thus, phosphoproteomic data could be useful for exploring phosphosites that are informative for predicting anti-tumor efficacy before response evaluation in clinical practice.

Our workflow enabled us to explore molecular variations in response to drug therapy that might contribute to acquired resistance. For example, upregulation of STAT5B (Y699) was found exclusively in the patient whose tumor did not respond to therapy. The observation was consistent with a previous report demonstrating an association between STAT5 phosphorylation with the response to anti-HER therapy^48^. We also found PAK4 (S474) hyperphosphorylation exclusively in the same patient. These phosphosites may play a role in acquired resistance to trastuzumab-based treatment via a bypass mechanism^42,43^. In addition, our analysis identified kinases that were activated after drug therapy for HER2-positive gastric cancer. Of note, the kinases that were activated exclusively in the patient without a PR might be considered as potential molecules for overcoming treatment resistance (e.g., RPS6KB2 [S6K2], CDK7, STK3, and STK39). RPS6KB2 (S6K2) and CDK7 activation were supported by phosphoproteomic data which revealed upregulation of phosphorylation at sites which mediate these kinases. CDK7 inhibitors have been under investigation in clinical trials^49^. CDK7 overexpression is associated with poor survival in patients with gastric cancer, whereas CDK7 inhibition decreases the proliferation in a gastric cancer cell line, suggesting CDK7 as a potential therapeutic target^50^. Of note, STK3 and STK39 activation were reported in HER2-positive breast cancer cell lines after anti-HER2 blockade, suggesting these roles in resistance to anti-HER2 therapy^51,52^. These results suggest the potential use of phosphoproteomic analysis to explore acquired resistance upon treatment and to identify therapeutic kinase targets in clinical practice.

Genomic or transcriptomic comparisons of tumor with non-tumor adjacent tissue may help with patient stratification in clinical trials^53,54^. In our study, kinases associated with the cell cycle (e.g., CDK2, CDK6) and the DNA damage response (e.g., ATM, ATR, CHEK2) were activated frequently in cancer, which is consistent with our previous research^23^. These results suggest that phosphoproteomic analysis of paired tumor and non-tumor adjacent tissue may also be clinically useful for patient stratification and/or target identification.

The advent of next-generation sequencing paved the road to personalized medicine and has been associated with several clinical breakthroughs^55,56^. Nonetheless, the majority of tumors lack ‘driver’ genetic alterations that present clear therapeutic opportunities^9^. We propose that phosphoproteomic profiling combined with bioinformatic analysis may help to expand personalized cancer management by identifying targets at the post-translational level. Large-scale comprehensive studies in conjunction with genomic analysis will provide the basis for the development of clinical proteogenomic approaches and the potentiation of personalized medicine in oncology. Our study suggested large phosphoproteomic variety after drug therapy, representing diversity of molecular responses in individual tumors. Considering the broad overlapping of the quantified phosphosites and proteins in the cell lines and clinical samples, our data indicate that cell line mixture will be highly useful as a reference for clinical proteogenomic analysis.

The major limitation of this study is the small number of samples collected in a single institution. Large-scale studies are needed to confirm the utility of our methods not only for personalized medicine based on pre-treatment samples but also to identify the optimal therapeutic approach for addressing post-treatment changes in molecular dysregulation. Given the highly inter-individual variation in kinase activity after drug therapy, further oncological annotation is required for the selection of potential therapeutic kinases in the clinical setting. We mainly used phosphoproteomic data for the identification of biological features in this study. However, integrated approaches such as proteogenomic analyses will offer deeper insights into cancer biology and will ultimately expand therapeutic options. Improvements to microscaled-proteomic workflows using clinical specimens are needed to realize these analyses. Current hypotheses should be tested in preclinical models and then confirmed in clinical studies. Furthermore, the absolute level of phosphorylation varies in a site-specific manner. The biological implications of perturbations at each phosphosite are of interest; however, the roles of the majority of phosphosites are largely unknown. Thus, further research is required to understand the phosphorylation-dependent regulation of proteins, including HER2.

Our data demonstrated the feasibility of mass spectrometry-based phosphoproteomic analysis with small biopsy specimens to track phosphorylation status in clinically relevant pathways over a treatment course. It is expected that phosphoproteomic analysis can be applied to find not only optimal therapy based on the pre-treatment samples but also combination therapy by adding other drugs or switching to new therapy adapting to the post-treatment changes in molecular dysregulation. This new dynamic treatment strategy using phosphoproteomic analysis will contribute to disease control and prolongation of patient survival.

## Conclusion

We show that highly sensitive phosphoproteomic analysis using endoscopic biopsy specimens is a clinically relevant tool for detecting dynamic molecular changes upon drug therapy. In addition, our data suggest that a phosphoproteomic analysis can be used to consecutively identify therapeutic targets in stage IV gastric cancer on an individual patient level.

## Materials and methods

### Clinical specimens

All patients were treated at the National Cancer Center Hospital (Tokyo, Japan). We collected endoscopic biopsy specimens from four patients with HER2-positive gastric cancer and four patients with HER2-negative gastric cancer. Post-treatment specimens from HER2-positive gastric cancer patients were obtained two months later after the initiation of drug therapy. In a single endoscopic procedure, three tumor specimens and three normal gastric mucosa specimens were collected concomitantly from each patient.

Each specimen was separately put in a screw-cap tube and immediately snap-frozen in liquid nitrogen within 20 s after collection. Frozen specimens were stored at -80 °C until further sample preparation.

All procedures involving human participants were performed in accordance with the ethical standards of the institutional and national research committee and with the Helsinki Declaration of 1964 and later versions. This study was approved by the Ethics Committees of the National Cancer Center Hospital (Tokyo, Japan) and National Institute of Biomedical Innovation Health and Nutrition (Osaka, Japan). Written informed consent was obtained from all patients. The date of protocol approval was 16 August 2017 at the National Cancer Center Hospital (Tokyo, Japan).

### Homogenization and solubilization of patient specimens

Each frozen specimen was transferred to a 1.5 mL tube supplied for use with a PowerMasher 2 (Nippi, Tokyo, Japan) and mixed with phase transfer surfactant (PTS) buffer supplemented with cOmplete™ protease inhibitor and PhosStop™ phosphatase inhibitor^57^. Each specimen was homogenized for 30 s, and subjected to boiling at 95 °C for 5 min. The cell lysates were further sonicated twice (15 min per set) with a Bioruptor sonicator (Cosmo Bio, Tokyo, Japan). The cell lysates were centrifuged at 20,000 rcf at 4 °C for 3 min and the supernatants of the corresponding three specimens were transferred to a new tube. After centrifugation, the protein concentration was measured with a detergent compatible (DC) protein assay kit.

### Protein precipitation, digestion, and desalting of clinical specimens

To remove contaminants, 500 µg of protein in each cell lysate was precipitated with methanol/chloroform precipitation. The pellets were re-suspended in PTS buffer and processed using a modified protocol from a previous study^23,57^. The amount of protein in the re-suspended solution was measured with a DC protein assay kit and 300 µg of each protein lysate was used for further steps. Then, the samples were reduced with tris (2-carboxyethyl) phosphine hydrochloride (TCEP) with a final concentration of 10 mM at 37 °C for 60 min, and alkylated with iodoacetamide (IAA) with a final concentration of 20 mM in the dark room at 37 °C for 30 min. Alkylation was terminated with L-cysteine with a final concentration of 21 mM at room temperature for 10 min. Each protein lysate was mixed with trypsin (protein weight: 1/50) and lysyl endopeptidase (protein weight: 1/50) and subjected to incubation for overnight at 37 °C. The samples were acidified with 1% trifluoroacetic acid (TFA) and centrifuged at 20,000 rcf for 10 min at 4 °C. Supernatants were desalted and separated into two tubes (99% for phosphoproteomic analysis, 1% for global proteomic analysis)^23^.

### Enrichment of phosphopeptides in clinical specimens

Enrichment of phosphopeptides with Fe^3+^-immobilized metal affinity chromatography (IMAC) resin was performed^23,58^. In this study, we enriched phosphopeptides on a stage-tip with Fe-IMAC resin. Briefly, a C^18^ disc was set in a 200-µL disposable tip, and Fe-IMAC resin was set on the C^18^ disc. Desalted peptides were passed through the IMAC/C^18^ stage-tip. Phosphopeptides were eluted with 60% acetonitrile (ACN) plus 0.1% TFA, and purified on the lower C^18^ disc.

### Preparation of cell line mixture as reference samples

MKN-28, MKN-45, MKN-74, NCI-N87, NUGC-3 and TMK-1 cells were cultured at 37 °C under 5% CO_2_. These gastric cancer cell lines were maintained in Roswell Park Memorial Institute (RPMI)-1640 supplemented with 10% fetal bovine serum (FBS). The gastric cancer cells were harvested after washing with ice-cold phosphate buffered saline (PBS) buffer containing PhosSTOP (phosphatase inhibitor) and cOmplete (protease inhibitor). Pellets of the collected cells were quickly frozen in liquid nitrogen and stored at − 80 °C prior to use. Lysis of the cell pellets was performed in PTS buffer supplemented with cOmplete protease inhibitor cocktail and PhosSTOP, and then all cell lysates were mixed. Protein concentration was determined using a DC protein assay according to the manufacturer’s protocol. A total of 8 mg of protein lysate (four lysates with 2.0 mg proteins) was reduced, alkylated, desalted, and 99% of elution was subjected to enrichment of phosphopeptides for reference samples for phosphoproteomic analysis and 1% of elution was used for reference samples for global proteomic analysis^59^.

### Tandem mass tag (TMT) labeling and fractionation of peptides on C^18^/SCX stagetip

TMT labelling for each sample was conducted using TMT 10-plex isobaric label reagent according to the manufacturer's protocol^23^. A correspondence table of each TMT label and each sample is summarized in Fig. S1. TMT-labelled peptides of each TMT batch were pooled and fractionated on a C^18^/SCX stage-tip^60^.

### LC–MS/MS analysis

LC–MS/MS was performed by coupling an UltiMate 3000 Nano LC system (Thermo Scientific, Bremen, Germany) and an HTC-PAL autosampler (CTC Analytics, Zwingen, Switzerland) to an Orbitrap Fusion Lumos mass spectrometer (Thermo Scientific)^61^. Peptides were delivered to an analytical column (75 µm × 30 cm, packed in-house with ReproSil-Pur C18-AQ, 1.9 µm resin, Dr. Maisch, Ammerbuch, Germany) and separated at a flow rate of 280 nL/min using a 145-min gradient from 5 to 30% of solvent B (solvent A, 0.1% formic acid [FA]; solvent B, 0.1% FA and 99.9% ACN)^61^. The Orbitrap Fusion Lumos mass spectrometer was operated in the data-dependent mode^61^. For phosphoproteomic analysis, survey full scan MS spectra (m/z 375 to 1,500) were acquired in the Orbitrap with 120,000 resolution after accumulation of ions to a 4 × 10^5^ target value^61^. Maximum injection time was set to 50 ms and dynamic exclusion was set to 30 s. MS2 analysis consisted of higher-energy collisional dissociation (HCD); automatic gain control (AGC) 1 × 10^5^; normalized collision energy (NCE) 38; maximum injection time 105 ms; 50,000 resolution and isolation window of 0.7 Da^61^. For global proteome analysis, survey full scan MS spectra (m/z 375 to 1500) were acquired in the Orbitrap with 120,000 resolution after accumulation of ions to a 1 × 10^5^ target value. Maximum injection time was set to 100 ms and dynamic exclusion was set to 10 s. MS2 analysis consisted of HCD; AGC 1 × 10^5^; NCE 38; maximum injection time 315 ms; 120,000 resolution and isolation window of 0.7 Da.

### Data processing for identification and quantification of peptides

The identification of proteins and phosphosites was carried out with MaxQuant 1.5.1.2 supported by the Andromeda search engine^62^. The reviewed amino acid database was obtained from the UniProt (human, release 2017_01) and combined with 262 common contaminants. Enzyme specificity was set to Trypsin/P (a C-terminal of Arg or Lys with the allowed cleavage at the proline bond). Miss-cleavages were tolerated up to two sites. Fixed modification was set as carbamidomethylation of cysteine residues. Methionine oxidation, serine, threonine, and tyrosine phosphorylation were assigned as variable modifications. False discovery rates of protein group, peptide-spectrum match (PSM), and posttranslational modification (PTM) site were less than 0.01. Peptides hit as “Reverse” or “Potential Contaminant” were not used in the following analysis. Only protein groups identified with at least two or more peptides (sum of razor and unique) were used in the further analysis. The cut-off criteria for localization probability at each phosphosite was greather than 0.75^63^. Peptide-Spectrum Matches (PSMs) were summarized in Table S11 [for phosphoproteomic analysis] and Table S12 [for global proteomic analysis]).

### Data processing for comparisons

Statistical analysis was carried out with Perseus 1.6.2.3^64^. We adopted two methods of normalization for group comparisons and individual comparisons using the quantitative data of TMT reporter ion intensities with log_2_ transformation. For the comparison of proteomic data between two different group (e.g., the pre-treatment HER2-positive gastric cancer group and the post-treatment HER2-positive gastric cancer group), the mean abundance value of a specific protein or phosphosite from the mixed cell line sample was subtracted from the corresponding value in each clinical sample and normalized by median centering of the values in each TMT channel. Next, batch correction was performed for TMT batches. In this study, proteins (or phosphosites) quantified at least one sample in each TMT batch (a total of four batches) were considered for subjects of imputation. Each minimum value was imputed to each protein (or phosphosite). Then, the batch effect was removed using the Combat function on sva package version 3.36.0. Using the corrected data, the mean quantitative value of protein (or phosphosite) of each group was calculated by averaging the quantitative values of clinical samples among the same group. Differentially expressed phosphosites with significance between two groups were selected if a phosphosite had log_2_ |FC|> 1 with a *p* < 0.05 for the Welch test (comparisons between the pre-treatment HER2-positive T group and the HER2-negative T group) or a paired *t*-test (comparisons between the post-treatment HER2-positive T group and the pre-treatment HER2-positive T group) of a difference between two groups. Differentially expressed phosphosites with tendency between two groups were selected if a phosphosite had log_2_ |FC|> 1 with a 0.05 ≤ *p* < 0.1 for the Welch test or a paired *t*-test of a difference between two groups. For individual comparisons between paired post-treatment and pre-treatment HER2-positive gastric cancer, the quantitative phosphosite value of each clinical sample was normalized by median centering of the values in each TMT channel. Differentially expressed phosphosites were selected if a phosphosite had log_2_ |FC|> 1.

### Principal component analysis (PCA)

To understand whether there were differences between the clinical samples, PCA was performed using the prcomp function (R environment version 4.0.2). The visualization of PCA was performed by ggplot2 package version 3.3.2.

### Phosphorylation profiling in the ErbB signaling pathway

Molecular information on the ErbB pathway was downloaded from KEGG^65^. Log_2_ transformed fold change (FC) values of each phosphosite were manually plotted on the picture of the pathway.

### Pathway enrichment analysis

Differentially expressed phosphosites with significance between two groups were subjected to the pathway analysis in WebGestalt using KEGG^66^. Pathways with a *p*-value < 0.05 were selected as significant pathways.

### Estimation of individual kinase activity

Kinase enrichment analysis on the ratio of quantitative values of phosphosites between two individual samples was performed using kinase-substrate enrichment analysis (KSEA)^67^. We selected kinases that had more than one kinase-substrate relationship as registered in PhosphositePlus^41^. A *p* value of less than 0.05 indicated that a kinase was significantly dysregulated.

## Supplementary Information

Supplementary Figures.

Supplementary Table S1.

Supplementary Table S2.

Supplementary Table S3.

Supplementary Table S4.

Supplementary Table S5.

Supplementary Table S6.

Supplementary Table S7.

Supplementary Table S8.

Supplementary Table S9.

Supplementary Table S10.

Supplementary Table S11.

Supplementary Table S12.
