# Supplementary material for: Temporal dynamics from phosphoproteomics using endoscopic biopsy specimens provides new therapeutic targets in stage IV gastric cancer
Source: Sci Rep. 2022 Mar 25;12:4419. doi: 10.1038/s41598-022-08430-7 (PMC8956597; doi:10.1038/s41598-022-08430-7)
Supplement: Supplementary file 1 — Supplementary Figures. [file 41598_2022_8430_MOESM1_ESM.docx]

**Type of the article: Original Article**

**Title: Temporal dynamics from phosphoproteomics using endoscopic biopsy specimens provides new therapeutic targets in stage IV gastric cancer**

Hidekazu Hirano, Yuichi Abe, Yosui Nojima, Masahiko Aoki, Hirokazu Shoji, Junko Isoyama, Kazufumi Honda, Narikazu Boku, Kenji Mizuguchi, Takeshi Tomonaga, Jun Adachi

The file contains

Figure S1–S5

**Figure S1** TMT labelling in this study and overlapping of quantified phosphosites/proteins between clinical specimens and cell line mixture


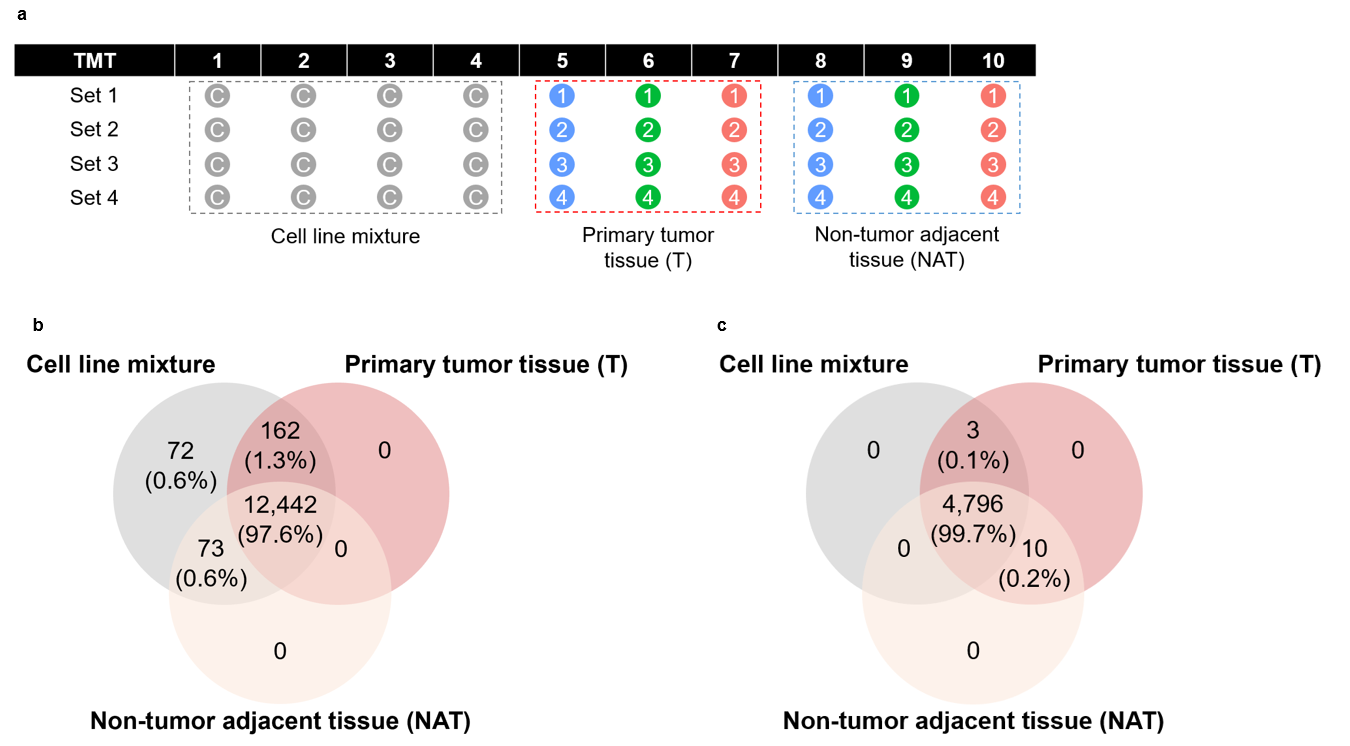


1. Details of TMT labelling in this study.

The number of each circle indicates patient number (blue [pre-treatment] and green [post-treatment] for patients with HER2-positive gastric cancer and red for patients with HER2-negative gastric cancer)

1. Overlapping of quantified phosphosites between the clinical specimens and cell line mixture

Numbers in the Venn diagram show unique and shared quantified phosphosites between cell line mixture samples, primary tumor tissues (T), and non-tumor adjacent tissues (NAT).

1. Overlapping of quantified proteins between clinical specimens and cell line mixture

Numbers in the Venn diagram show unique and shared quantified proteins between cell line mixture samples, primary tumor tissues (T), and non-tumor adjacent tissues (NAT).

**Figure S2** Summary of the global proteomic results


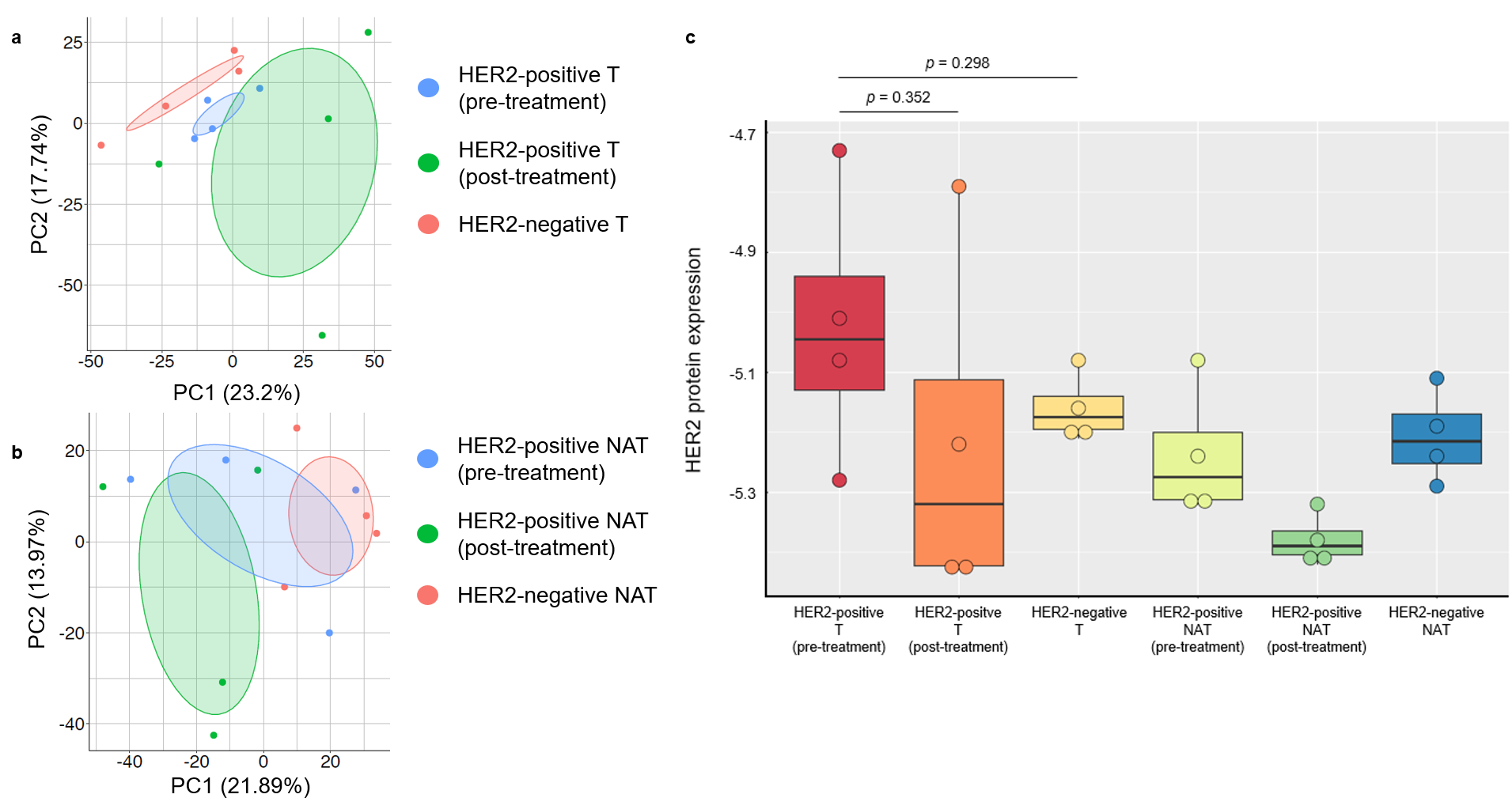


a. Principal component analysis of tumor samples using global proteomic data.

b. Principal component analysis of non-adjacent tumor samples using global proteomic data.

c. Boxplots show the distribution (median, 25th and 75th percentile) of HER2 protein expression in each sample group.

**Figure S3** Pathway analysis for comparing phosphoproteomic data between pre-treatment HER2-positive gastric cancer and HER2-negative gastric cancer


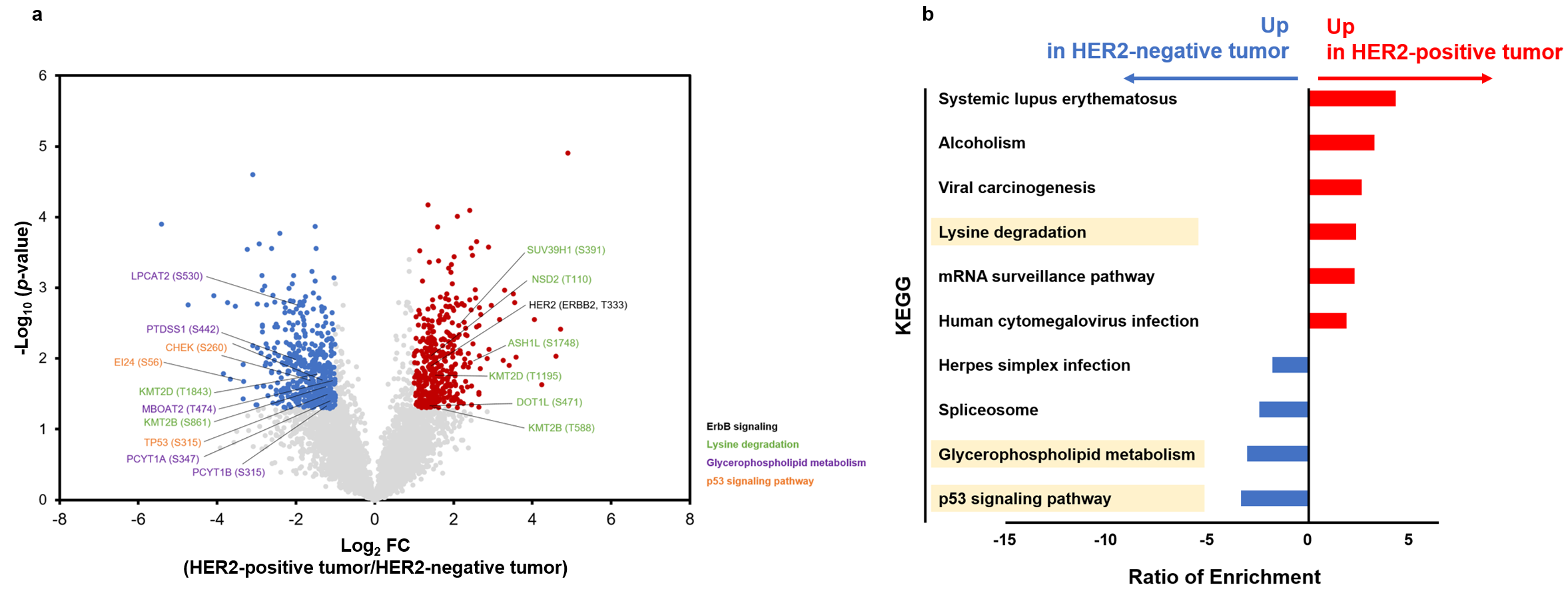


a. A volcano plot of the log_2_ fold change (FC) in each phosphosite and the -log_10_ of the *p*-values. Phosphosites with significant upregulation in pre-treatment HER2-positive gastric cancer and those with significant upregulation in HER2-negative gastric cancer are shown as red and blue circles, respectively. Phosphosites without significant differences are shown as grey circles. Phosphosites in noted pathways are labeled in different colors.

b. Pathway analysis based on the pathway information in KEGG. Pathways with a *p*-value < 0.05, representing enrichment of the phosphosites that are upregulated in pre-treatment HER2-positive gastric cancer are shown as red bars, while those of phosphosites that are upregulated in HER2-negative gastric cancer are shown as blue bars.

**Figure S4** Pathway analysis for comparing the phosphoproteomic data between post-treatment HER2-positive gastric cancer and pre-treatment HER2-positive gastric cancer


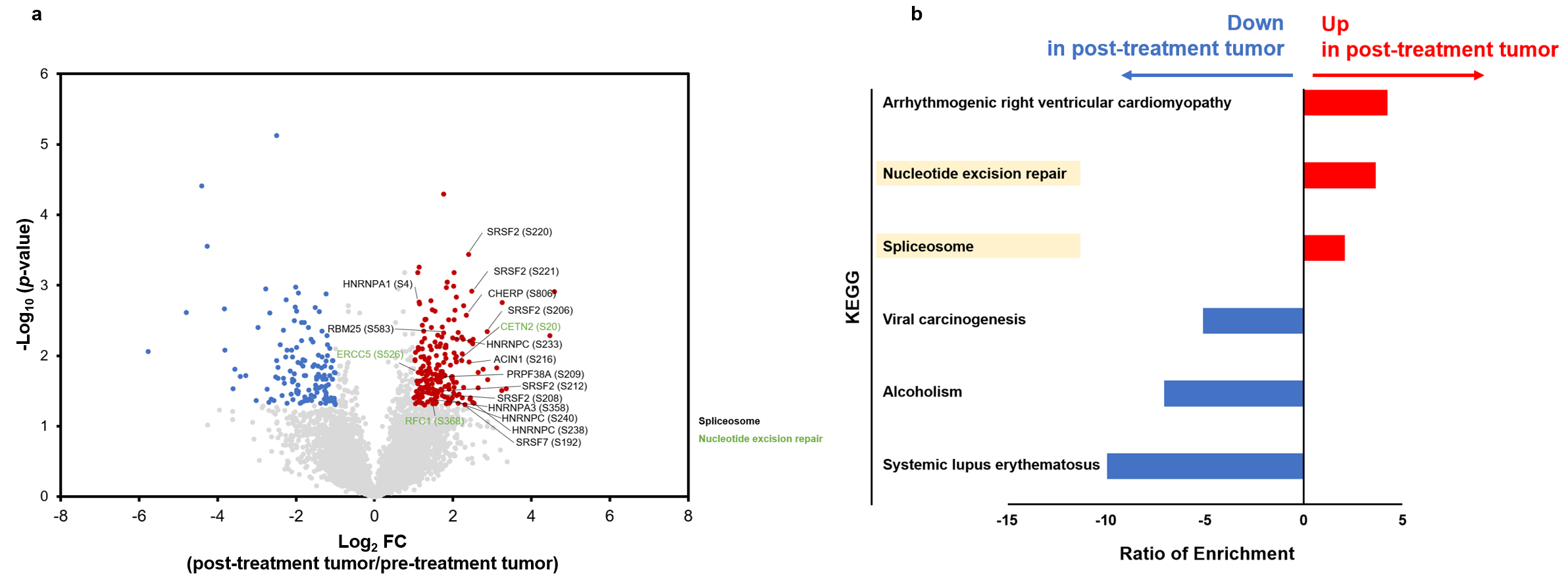


a. A volcano plot of the log_2_ fold change (FC) in each phosphosite and the -log_10_ of the *p*-values. Phosphosites with significant upregulation and downregulation in post-treatment HER2-positive gastric cancer are shown as red and blue circles, respectively. Phosphosites without significant differences are shown as grey circles. Phosphosites in noted pathways are labeled in different colors.

b. A pathway analysis based on the pathway information in KEGG. Pathways have *p*-value < 0.05, representing enrichment of the phosphosites that are upregulated in post-treatment HER2-positive gastric cancer are shown as red bars, while those of phosphosites that are downregulated are shown as blue bars.

**Figure S5** Upregulated phosphosites mediating kinase activity after treatment in HER2-positive gastric cancer


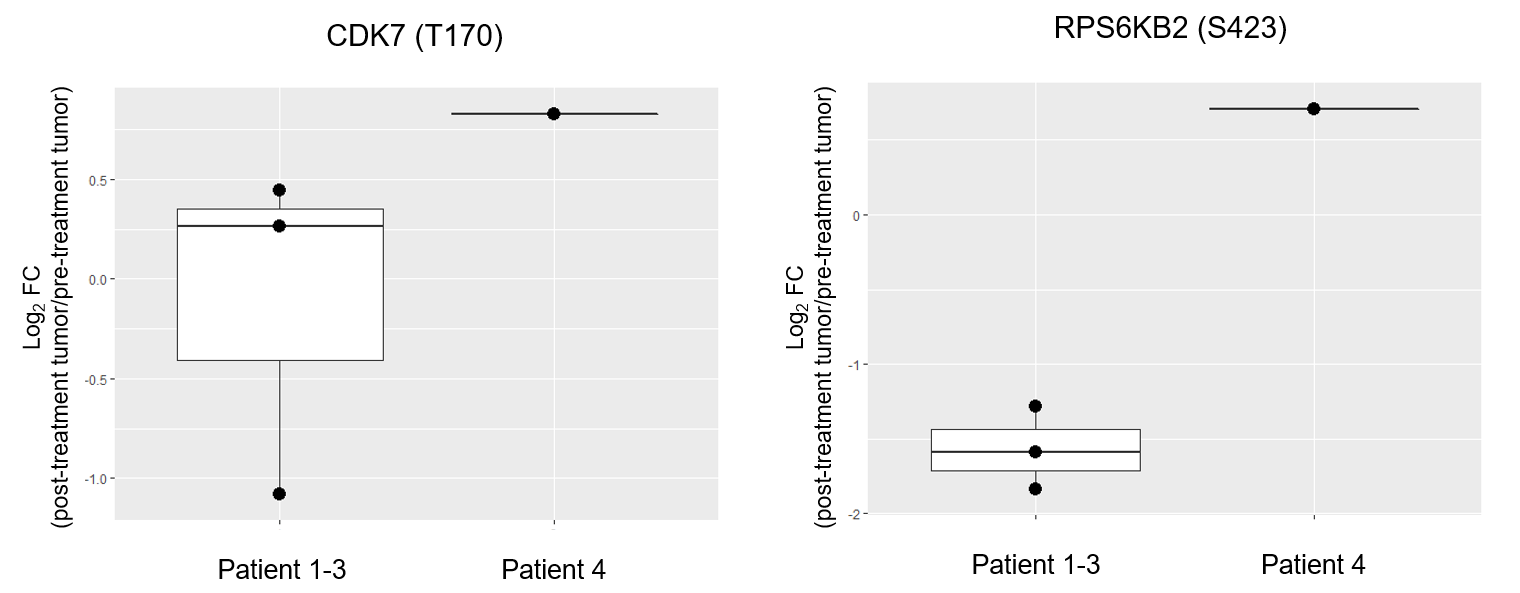


Boxplots show the distribution (median, 25th and 75th percentile) of log_2_ fold change (FC) (post-treatment tumor/pre-treatment tumor) of phosphorylation in the Patients 1–3 group.
